# Three-Dimensional Electromagnetic Torso Scanner

**DOI:** 10.3390/s19051015

**Published:** 2019-02-27

**Authors:** Sasan Ahdi Rezaeieh, Ali Zamani, Konstanty S. Bialkowski, Graeme A. Macdonald, Amin M. Abbosh

**Affiliations:** 1 School of Information Technology and Electrical Engineering, The University of Queensland; St. Lucia, Brisbane, Queensland 4072, Australia; a.zamani@uq.edu.au (A.Z.); ksb@itee.uq.edu.au (K.S.B.); a.abbosh@uq.edu.au (A.M.A.); 2 PA-Southside Clinical School, The University of Queensland; St. Lucia, Brisbane, Queensland 4072, Australia; g.macdonald@uq.edu.au; 3 Translational Research Institute and Department of Gastroenterology and Hepatology, Princess Alexandra Hospital, Woolloongabba, Brisbane, Queensland 4102, Australia

**Keywords:** three-dimensional torso scanning, electromagnetic imaging, thoracic diseases

## Abstract

A three-dimensional (3D) electromagnetic torso scanner system is presented. This system aims at providing a complimentary/auxiliary imaging modality to supplement conventional imaging devices, e.g., ultrasound, computerized tomography (CT) and magnetic resonance imaging (MRI), for pathologies in the chest and upper abdomen such as pulmonary abscess, fatty liver disease and renal cancer. The system is comprised of an array of 14 resonance-based reflector (RBR) antennas that operate from 0.83 to 1.9 GHz and are located on a movable flange. The system is able to scan different regions of the chest and upper abdomen by mechanically moving the antenna array to different positions along the long axis of the thorax with an accuracy of about 1 mm at each step. To verify the capability of the system, a three-dimensional imaging algorithm is proposed. This algorithm utilizes a fast frequency-based microwave imaging method in conjunction with a slice interpolation technique to generate three-dimensional images. To validate the system, pulmonary abscess was simulated within an artificial torso phantom. This was achieved by injecting an arbitrary amount of fluid (e.g., 30 mL of water), into the lungs regions of the torso phantom. The system could reliably and reproducibly determine the location and volume of the embedded target.

## 1. Introduction

Clinical imaging systems, particularly ultrasound (US), computerized tomography (CT), and magnetic resonance imaging (MRI) are essential for contemporary medical practice. They are used to diagnose disease, and monitor disease progression and response to therapies. US, CT and to a lesser extent MRI can be used to guide diagnostic procedures such as biopsy, and in the therapeutic setting to drain pathological fluid collections and to deliver treatments with great precision to targeted regions of the body. Despite revolutionizing many areas of medicine, all these modalities have limitations. Ultrasound requires significant training and expertise in order to reliably obtain accurate images. CT and MRI require significant shielding. For CT, this is to limit exposure to potentially harmful ionizing radiation. MRI requires shielding so that the strong magnetic fields employed do not cause problems in the vicinity of the scanner. This shielding limits the portability of these devices and they are generally only found in fixed settings. It also adds to the cost of installing the equipment. Safety can be another issue: CT is one of the most widely used diagnostic tools and yet patients are exposed to ionizing radiation, albeit with the potential benefit of accurate diagnosis. While these imaging systems represent a major advance in medicine, there is still room for improvement for imaging systems in terms of safety for patients and staff, portability, ease of use, and cost. Ideally, improvements in these four domains would potentially allow for expansion in the indication for use, such as expanded use in screening and surveillance programs; or in more frequent monitoring of critically ill patients; and for monitoring response to clinical interventions.

Therefore, there is a sensible necessity in developing new medical imaging modalities to address these issues. Electromagnetic imaging has been proposed in recent years as a promising technique that can provide two- or three-dimensional images, to identify a range of pathologies within a desired imaging domain [1,2,3,4,5,6,7,8]. These systems primarily operate based on the fact that pathological processes, such as cancer, have different dielectric properties from healthy tissue [9,10]. These differences occur because of changes in the water, lipid and mineral composition of diseased tissues, and the coexistence of related processes, such as inflammation and fibrosis, which lead to a change in the dielectric properties compared to healthy tissue. Thus, the characteristics of a reflected or transmitted electromagnetic signal, such as its amplitude and/or phase, from diseased tissue are likely to be significantly different from normal/healthy tissues [8].

Electromagnetic imaging has been purposed for many medical applications, such as the detection of breast cancer or brain stroke, torso scanning to recognize heart failure, etc. The breast imaging systems proposed in [11,12] utilize an array of monopole antennas around the breast, and a confocal imaging technique or microwave tomography to process the gathered signals and form images. The imaging system presented in [13] utilizes microwave pulses to obtain information from deeply situated breast malignancies by analyzing acoustic waves gathered using transducer array. To improve the constructed images in electromagnetic imaging algorithms, a laser system that operates as a surface estimator of the imaged object is utilized in [14]. A differential imaging system is proposed in [15] where two arrays of similar antennas are placed around both breasts. This system is based on the fact that the scattering profile of left- and right-side breasts are roughly similar to each other and the premise that the probability of synchronic malignancies in both breasts is less than 10%. Thus, by comparing the scattered profile of two breasts at the same time, any abnormality in one side would result in big variations in the reflected signals, and thus cancer can be detected.

EMI has also been utilized in head imaging. A simple system, which uses a single antenna rotating around the head and a fast frequency imaging algorithm, is proposed in [16] to identify tumors inside the head. To accelerate data acquisition, a different configuration that uses an array of antennas is proposed in [17]. Recent attempts have resulted in the proposal of a microwave helmet to diagnose intracranial bleeding [18]. The signals captured by the eight antennas of the system are statistically analyzed and compared to a large database to classify strokes. 

Considering the volume of the torso and the diversity of tissues and pathologies that occur in the chest and abdomen, multiple methods have been trialed to identify disease in the torso. One focus has been on identifying pulmonary edema (fluid accumulation inside the air spaces and tissues of the lungs), because this is one of the primary signs of congestive heart failure. An early approach was to study the changes in the amplitude and/or phase of the reflected signal from the torso, or transmitted through the chest [19,20]. Bio impedance spectroscopy is another method where the body is modeled as an electric circuit, and each tissue is assigned an impedance [21]. Accumulation of water inside the lungs alters their impedance, and assists in the early recognition of a clinical issue. Ultra-wideband radar is also utilized to identify the abnormal accumulation of fluid in tissues [22]. This technique relies on the fact that electromagnetic signals have stronger reflections from fluid compared to the surrounding tissues, such as lungs and heart. In that system, the reflected signals are compared to a reference signal from a healthy individual. 

One of the advanced techniques that has been developed recently is the estimation of dielectric properties inside an imaged domain [23]. In this technique, a training algorithm is used to estimate the dielectric properties of imaged objects. Once the dielectric properties of the healthy/normal objects are obtained, they are used as a reference to detect any changes in the dielectric values due to any abnormality. 

As can be realized from the abovementioned systems, electromagnetic imaging systems mainly use an antenna or array of antennas or sensors to transmit/receive electromagnetic signals to/from the human body. These signals are then processed using various signal processing and computational electromagnetic techniques to form two- or three-dimensional images. One of the main problems in the design of the hardware elements, e.g., antennas, for these systems is the size of the antennas. This is because these systems mainly operate at lower microwave frequencies of about 1 GHz, resulting in long wavelengths that are proportional to the large and bulky size of the expected antenna designs. This issue has been tackled using different methods, such as folding the antennas structure to form a three dimensional shape [7], use of fractal structures to increase the electrical length of the antenna [24], employing phased array structures [25], utilizing dielectric resonator antenna configurations with high permittivity materials [26], and combining loop and dipole structures to achieve a wide operating bandwidth and a unidirectional radiation [27]. Another major issue has been the effect of skin reflection on electromagnetic signals. Several imaging algorithms have been proposed to tackle the effect of skin reflection removal from the scattered signals [28], estimating the surface of the imaged domain [2] in addition to boundary estimation of the imaged domain [29]. The evolution of these techniques has led to the fabrication of electromagnetic imaging tools that are being used for breast imaging [30,31] and head imaging systems [32].

The torso is a clinically important region because it hosts organs such as the heart, lungs, liver and kidney, of which their related diseases are among the leading causes of death in the world [33]. Recent efforts have led to proposals for two torso scanning systems [34,35]. The first system [34] is comprised of an array of sixteen antennas that are embedded inside a hospital bed and scans the posterior of the torso adjacent to the array. The second system [35] utilizes two elliptical arrays of antennas that are located at fixed locations inside an elliptically shaped case. Analyzing existing electromagnetic imaging systems reveals that while these systems provide significant innovations, they suffer from either hardware or software limitations. Some of those systems require the use of coupling medium [11,12,13] and external elements to provide penetration and estimate shape of the image [14]. This adds to the complexity of the design, weight of the system and its portability. Almost all existing systems rely on a priori information regarding the healthy state/profile of the imaged domain as a reference to help identify pathology in imaging [11,23,34,35]. While this can be achieved in theory, it is an impractical approach from a realistic application point of view. Thus, the majority of the proposed techniques/systems would not be viable if the healthy state of the patients were not available. 

To address this problem, we propose a global scanning mechanism that can obtain information from all angles and areas along the torso to generate three-dimensional (3D) images. The system is comprised of an array of fourteen antennas that are co-located on a flange that is fabricated in an elliptical shape similar to the cross-sectional shape of the torso. To scan the whole torso, the flange is mechanically moved using a stepper motor, located within a microwave-transparent case. To fully utilize the scanning capability of the proposed scanner, a 3D imaging algorithm is also proposed. This algorithm utilizes a radar-based microwave imaging technique to compute two-dimensional image data for each slice in front of the array. The computed data from all slices of the scanned torso are then interpolated to obtain three-dimensional images showing the location of the various organs and any associated pathology in the region of interest. Unlike the available methods, this algorithm does not require prior data from an individual to detect the location and size of any accumulated fluid. To verify the capabilities of the proposed system and the imaging algorithm, a model of pulmonary abscess was developed, and the system was tested on its ability to identify 30 mL of water inside a torso mimicking phantom. The system was successful in detecting the location, size and shape of the fluid. With its novel structure and imaging algorithm, the designed system demonstrates significant advantages over the previously presented electromagnetic torso scanner [35]. Previous torso scanners consist of two fixed arrays of antennas that can only scan two specific positions alongside the torso, e.g., superior and inferior lungs. Thus, they cannot accurately locate the fluid or any abnormality, such as tumor in the chest area, except for positions in front of the array. The proposed system includes a movable flange that can scan any position alongside the torso and can be utilized to diagnose a range of pathologies. Moreover, due to the design of new generation of antennas, this system utilizes a higher number of antennas, which improves the accuracy of detection. As a consequence of the three-dimensional scanning capability, the proposed system is capable of identifying the shape and spatial location of a disease process, something not possible using existing systems. 

## 2. Proposed System

**Figure 1:**
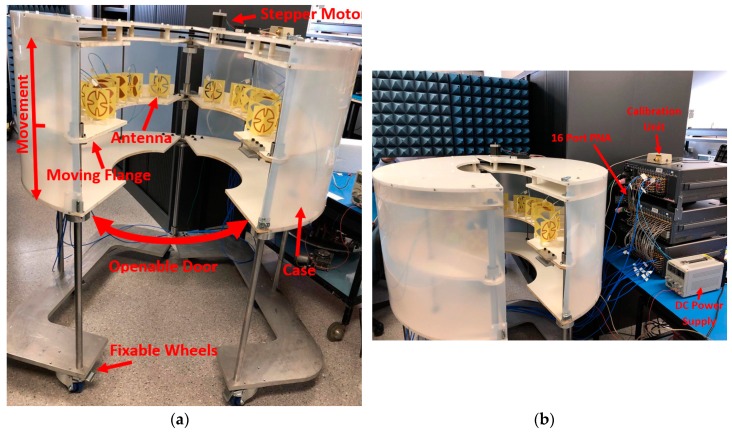
(**a**) Proposed 3D torso scanning system and (**b**) its testing environment.

The proposed electromagnetic torso scanner is shown in Figure 1. The system is comprised of an array of 14 antennas that are located on a movable flange and covers the torso area. This configuration is adapted to conform to the shape of the torso and obtain signals from each angle around the chest and upper abdomen. In this system, the subjects stand inside the designed cavity at the center of the machine, and images are obtained in the transverse plane. The flange is designed in two pieces that each host seven antennas. This configuration is adopted to allow for the system to be easily opened to accommodate the patients. The flange is connected to a stepper motor using four rods that are connected to its structure on either half of the system. Using a series of plastic belts and bolts, both sides can be simultaneously displaced vertically with an accuracy of a fraction of 1 mm. The stepper motor is controlled using a variable DC power unit. The system is located on a base that is supported by wheels to facilitate portability of the system in a clinical or lab environment. To reduce the effect of medium in the performance of the antennas, the main case and parts of the system are fabricated using plastic and PVC material with low permittivity. However, to achieve a sturdy design, the supporting elements are fabricated using metals. Special attention is paid to locate these metallic elements either far from the antenna element or behind the antenna structure. The antennas are then connected to a 16-port multi-port Vector Network Analyzer (VNA) formed using M9485A interface cards from Keysight. The utilized VNA is calibrated for all the required ports up to the end of the coaxial cables, thus removing the effect of cables and the network analyzer in the performance of the system. The whole process is controlled using a laptop that is connected to the VNA using a network cable (not shown). 

## 3. System Elements

The proposed 3D torso scanner is comprised of two main parts: (1) Hardware (antennas) and (2) Software (imaging algorithm). This section will discuss the requirements of these units and the proposed and utilized techniques.

### 3.1. Antenna Design

**Figure 2:**
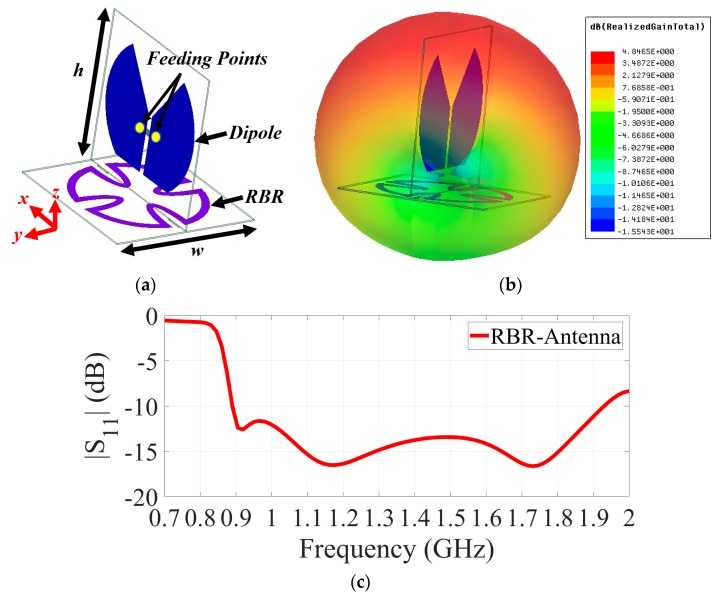
(**a**) Utilized RBR antenna [39] *w* = *h* = 100 mm. (**b**) Three-dimensional radiation pattern of the RBR antenna at sample frequency of 1 GHz and (**c**) its |S_11_| plot.

As realized, the antennas are the key elements of the hardware system that transmit and receive the signals to and from the torso. According to previous studies, a wide operating bandwidth of around 1 GHz provides the required penetration for the transmitted signals, while providing a reasonable resolution for the resultant image [36]. Electromagnetic imaging systems generally require compact, wideband and unidirectional antennas [37]. To satisfy the requirements of the system, a resonance-based resonator (RBR) antenna is utilized in this system. The proposed antenna is depicted in Figure 2a, with its details explained in [38]. The antenna is comprised of a meandered loop antenna and a half-wavelength dipole antenna that has a compact size of 0.22λ × 0.22λ, where λ is the wavelength at the lowest resonance. The loop structure both acts as a resonator and a reflector at the resonance frequency of about 1 GHz, and hence creates a wide operating bandwidth at 0.83–1.9 GHz (79%) (Figure 2b). It has a peak gain of 3.8 dBi with a unidirectional radiation across the operating bandwidth, with peak front-to-back-ratio (FBR) value of 9 dB. A 3D representation of the pattern of the antenna for a sample frequency of 1 GHz, where the antenna radiates most of its received power in the z-direction, is shown in Figure 2c. A comprehensive study in [39] revealed that this structure is capable of reducing surface wave propagation, hence enabling the positioning of a large number of antennas besides each other without affecting antenna performances.

**Figure 3:**
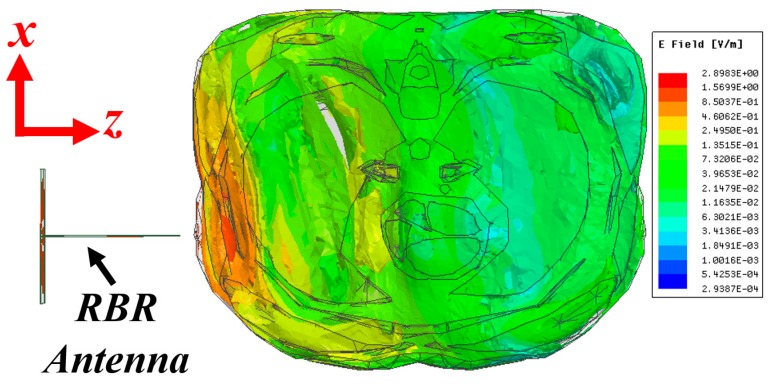
Calculated electric field density at sample frequency of 0.9 GHz.

To verify the effectiveness of the utilized antenna in providing the required signal penetration into the torso, a simulation is conducted using Ansys Electronic Desktop 2018. The simulation is performed on a virtual computational model of human torso that is imported to High Frequency Structure Simulator (HFSS). It includes all internal organs with a resolution of 2 mm. To reduce the computational cost and present a clear demonstration of the electric field distribution inside the torso, a cross-section of the model with thickness of 10 cm is cut and utilized. As seen from Figure 3, the intensity of the electric field distribution is stronger at outer tissues, such as skin, muscle and fat, and decreases with the distance, yet there is a reasonable penetration to the center of the torso. This is the main motivation behind the design of an elliptical system, where the antennas are located at different angles around the torso to obtain a strong scattered field from different regions. Nevertheless, it proves that the proposed antenna is capable of directing the electromagnetic energy inside the torso area.

### 3.2. Safety Criteria

**Figure 4:**
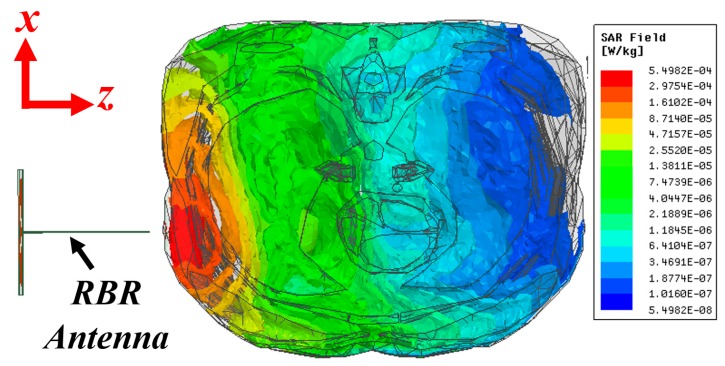
Calculated SAR value at sample frequency of 0.9 GHz using 1 mW transmission power.

One of the critical requirements of an electromagnetic imaging scanner system is the safety of the utilized system, measured using the specific absorption rate (SAR). SAR defines the amount of absorbed electromagnetic energy by tissues exposed to an electromagnetic wave/field. To measure the amount of SAR inside the torso, a similar simulation setup for calculation of electric field is utilized, and the SAR values are calculated and presented in Figure 4. During this test, a power level of 1 mW or 0 dBm is utilized. This is the amount of power that will be utilized during practical measurements. The obtained maximum SAR from the simulation is 0.0054 W/Kg. This occurs in the skin and regions of the body closest to the antenna. This value is well below the acceptable power level of 1.6 W/Kg defined by the Federal Communications Commission (FCC) Office of Engineering & Technology [39]. Based on this simulation, the system appears safe for testing on human subjects.

### 3.3. Imaging Algorithm

To interpret the collected electromagnetic signals and produce clinically meaningful images of the scanned torso, a three-dimensional imaging algorithm has been developed. In this algorithm, the recorded data are firstly calibrated by subtracting the measured complex electric fields (S-parameters) in the absence of phantom $E_{mn}^{e}$ from electric fields in the presence of the phantom $E_{mn}^{p}$ to eliminate measurement errors due to environment noises and inevitable antennas fabrication and assembly errors:

(1) $$\begin{array}{cc} E_{mn}^{c}=E_{mn}^{p}-E_{mn}^{e} & m,n=1:Na \end{array}$$

where $E_{mn}^{c}$ represents the calibrated data, *m* and *n* are receiver and transmitter indices, and *Na* represents the number of imaging antennas. 

Due to the contrast between dielectric properties of the skin and the air in front of the antenna, a large portion of the wave is reflected back to the antenna. These reflections are typically strong enough to mask signals from deep tissues. Hence, reflections from the outer layers should be removed or considerably mitigated to allow for accurate assessment of tissue composition within the imaged object. To that end, an average subtraction method [40] is used. In this technique, at each frequency, the average value of the calibrated signals is subtracted from each signal. Then, it is used to compensate for the effect of signal clutter caused by strong reflections from the skin. The process is applied on the calibrated reflection ($E_{nn}^{c}$) and transmission signal ($E_{mn}^{c}$) separately. For each type, the average of all received signals is subtracted from the related antenna’s signal at each frequency step:

(2) $$E_{mn}^{r}=\{\begin{array}{c} E_{nn}^{c}-\frac{1}{N_{a}}\sum_{n=1}^{Na}E_{nn}^{c}\\ E_{mn}^{c}-\frac{1}{N_{a}(N_{a}-1)}\sum_{\begin{array}{l} m,n=1\\ m\neq n \end{array}}^{Na}E_{mn}^{c} \end{array}$$

where $E_{mn}^{c}$ represents the clutter-removed signal.

**Figure 5:**
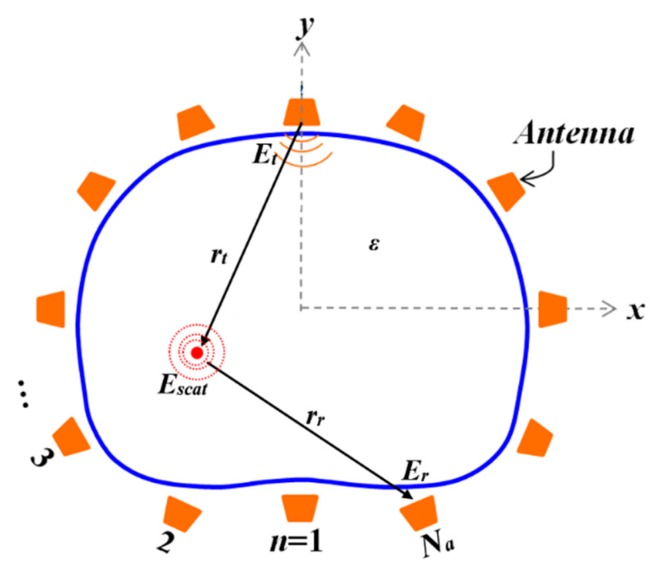
Imaging domain.

It should be noted that the skin has different effects at different frequencies. Hence, this technique is applied to the signals received by different antennas at each frequency sample to filter out those effects. In addition, the system is designed to have the antennas perpendicular to the skin and at the same distance. Thus, they will receive almost similar skin reflections, making those reflections the main contributor to the average signal value. This will enable the clutter removal technique to eliminate the effect of strong skin interface reflections with negligible effect on the target response. The calibration and clutter removal preprocesses are performed on the obtained data from each scanned slice. The cleaned data of each slice are then processed by a modified version of the fast frequency-based imaging algorithm [41] to compute the power intensity of scattered waves on the *xy* plane in front of each slice at height *z*:

(3) $$I(x,y;z)={\sum_{f=1}^{N_{f}}\left|\sum_{m=1}^{N_{a}}\sum_{n=1}^{N_{a}}E_{mn}^{r}(f;z)J_{1}(kr(x,y))e^{-i(kr(x,y))}\right|}^{2}$$

where *N_f_* is the number of frequency samples; ***J***_1_ (***kr***) is the Bessel function of the first kind first order; ***k*** is the complex average wavenumber of the imaging domain including the air and all tissues in the wave propagation path in front of antennas; *r* (*x*, *y*) = *|**r_t_*** (*x*, *y*)*| + |**r_r_*** (*x*, *y*)*|* is the distance from receiver to the scatterer to the transmitter as shown in Figure 5; and *f* is considered to be the frequency step indices. In the modified algorithm, the square of the sum of electric fields from different antenna locations are calculated to estimate the power intensity of electric field at each frequency step. Due to the different penetration capability of different frequencies, the estimated power intensities from different frequency steps are summed together to enhance detection accuracy of the algorithm. It is worth noting that the Bessel function ***J***_1_ (***k****r*), and the exponential part in Equation (3) do not change by *z*, and hence are calculated once and reused for the calculation of power intensity of all slices. 

The computed power intensities from all scanned slices can be mapped to a three-dimensional volume to create the final image; however, due to the relatively large scanning step (1 cm) in *z* direction for a faster scanning process, the direct image construction will significantly reduce the image resolution. Therefore, a slice interpolation technique [42], in which new intervening slices are estimated without changing the level of discretization within the slice itself, is utilized to improve the three-dimensional image resolution. In that regard, the intensity of a voxel *v^q^* = (*x*, *y*, *z^q^*) is estimated from the intensities of the voxels in lower and upper levels, *v^q−^*^1^ and *v^q+^*^1^, respectively:

(4) $$I^{\prime }(v^{q})=\frac{\left\|v^{q+1}-v^{q}\right\|}{\left\|v^{\left(q+1\right)}-v^{\left(q-1\right)}\right\|}I(v^{\left(q-1\right)})+\frac{\left\|v^{\left(q-1\right)}-v^{q}\right\|}{\left\|v^{\left(q+1\right)}-v^{\left(q-1\right)}\right\|}I(v^{\left(q+1\right)})$$

where ‖ ‖ denotes Euclidean distance between voxels.

The interpolated image shows the intensity of strong scatterers inside the three-dimensional imaging region. Therefore, the obtained image can define the location of the water, which has considerably higher dielectric properties compared to surrounding healthy tissues inside torso.

## 4. Experimental Verification

**Figure 6:**
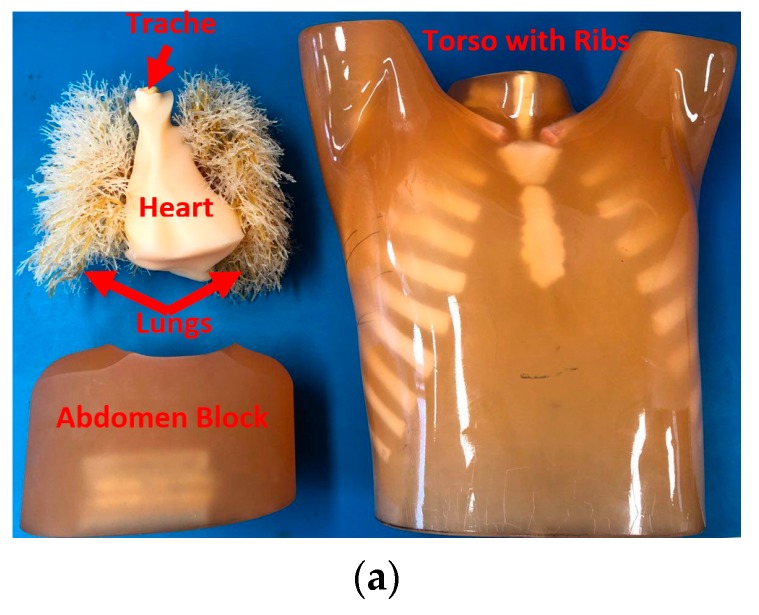

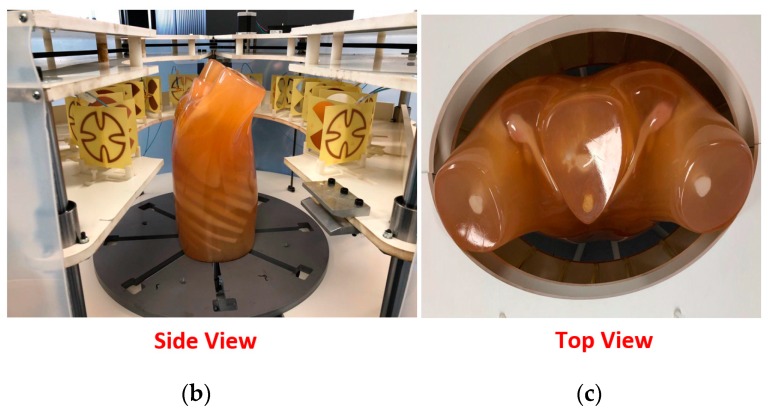
(**a**) Utilized torso phantom, and positioning of the phantom inside the scanner: (**b**) side view and (**c**) top view.

To verify the applicability of the proposed three-dimensional torso imaging system and the imaging algorithm, its performance was tested using a realistic size torso phantom. Due to ease of simulation, a pulmonary abscess model was selected for the validation process. Pulmonary abscess happens when a local inflammation leads to accumulation of fluid inside lungs. The utilized phantom, which is shown in Figure 6a, includes embedded synthetic bones, which are built using epoxy resin, and four internal organs including heart, lungs, abdomen and trachea that are built using polyurethane (gravity 1.06) with different mixtures. It has a size of 43 × 40 × 48 cm^3^, with a chest circumference of 94 cm. The chest wall thickness was based on clinical data from the company KyotoKagaku [43]. The measured permittivity of the materials used to emulate the fat and bone were between 3.3–2.9 and 3.5–3 at 0.9–2 GHz, respectively, while their measured conductivity was between 0.17–0.32 (s/m). The measured relative permittivity of human bone cortical and fat are around 12.4 and 5.4 at 1 GHz, respectively [44]. Thus, the phantom only roughly emulates the dielectric properties of organs inside the torso. Yet, this phantom is utilized due to its possessing a structural complexity that is analogous to that seen in the human torso. Thus, the capability of the imaging algorithm can be tested against the reflections from the different tissue boundaries. 

**Figure 7:**
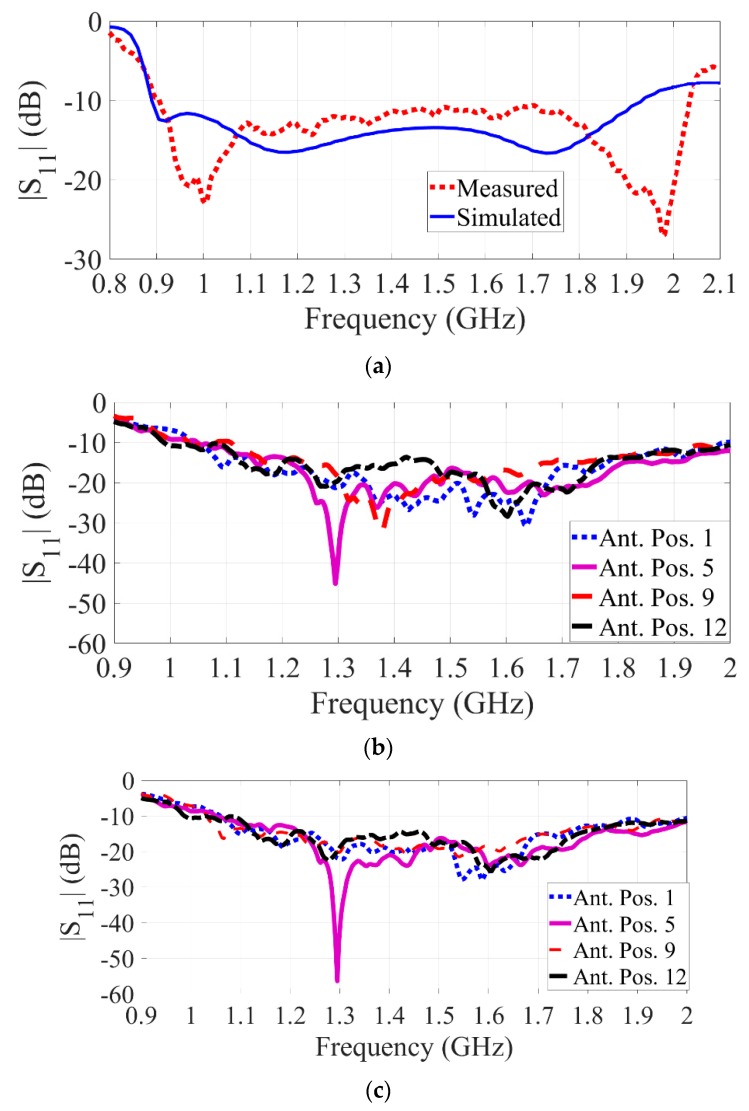
|S_11_| performance of the (**a**) isolated single antenna after VNA calibration; and |S_11_| performance of the antenna in the array in the integrated system for four arbitrary positions: (**b**) when the imaging domain is empty, and (**c**) when unhealthy case is present.

To run the tests, the phantom is located on an acrylic holder at the opening of the system (see Figure 6b) with its height adjusted so the scanning device can image the whole chest using the moving mechanism. As seen from Figure 6c, when the door is closed the phantom is closely spaced inside the scanning area with an average antenna to torso distance of 1 cm. The fact that the head of the phantom/patient is located out of the scanner area is an advantage of the proposed system compared to imaging tools such as MRI. This reduces the claustrophobia experienced by some subjects when they need to remain inside a closed cavity for image acquisition. To scan the whole torso, the flange is moved to 23 different positions, obtaining information from each corresponding layer and angle around the torso. It should be noted that the number of positions can be adjusted depending on the required accuracy and application of the system. A laptop is used to coordinate the measurement process, which involves communicating to both the multi-port VNA as well as the motor controller. To calibrate the system from the perspective of the VNA and cables, a full Open-Short-Load-Thru calibration for all 196 S-parameters (corresponding to each combination of reflection and transmission) might be performed weekly or monthly, depending on the requirements of the clinical tests. The motor controller, which is an Arduino microcontroller running “GRBL”—a high-performance CNC controller using pure C—interfaces the computer to the stepper motor using USB and a stepper driver, respectively. At each image acquisition position of the flange, the multi-port VNA undertakes a complete measurement from each antenna to every other antenna, which for the 14 antennas gives a total of 196 transmission and reflection parameters. Each of these parameters contains frequencies between 500 MHz and 2 GHz with 400 points, performed using an IF bandwidth of 1 kHz. The selected IF bandwidth means that over 100 dB of dynamic range is obtained for each measurement point. To complete a measurement, the motors are moved, and then a measurement sweep is initiated, the total time per position is around 12 s. This corresponds to a complete torso scan taking around 5 min to perform. This compares favorably with the time required to acquire CT or MRI images. The received signals by VNA are recorded in a processing unit, e.g., a laptop, and are processed using the proposed 3D imaging algorithm. Two calibration mechanisms are utilized before processing can be done. Initially, each antenna is calibrated using the calibration kit of the utilized VNA to compensate for the effect of the system and cable losses. The measured response of the single isolated antenna is presented in Figure 7a and is compared to the simulated results. The existing discrepancy is due to fabrication errors and additional effect of flange. Yet, the antenna operates within a designed operating frequency of 0.9–2.03 GHz. Secondly, free space scans are performed when no object is located in the imaging domain. In this case, the calibration is done by measuring an empty domain for all 23 positions of the movement system, for all of the antennas. This process should be performed before each human scan. The obtained raw signals after this calibration for the utilized antennas at four different positions, which are part of the 14-antenna array, are depicted in Figure 7b. As can be seen, the antenna operates within the designed frequency band (Figure 7a) with slight shift at the low frequency towards 1 GHz. This shift is due to effect of passive elements in the system. Nonetheless, the antenna provides the wide operating bandwidth required at different positions. 

**Figure 8:**
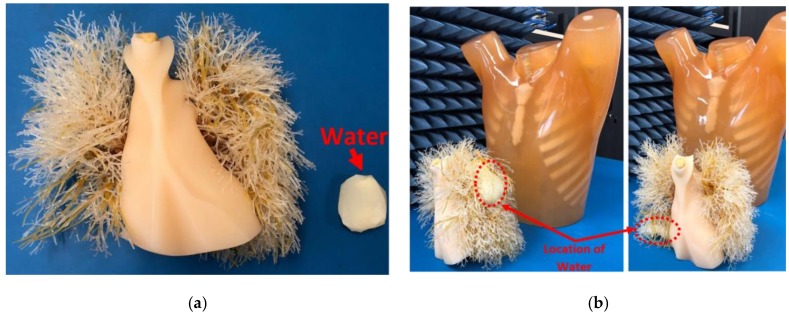

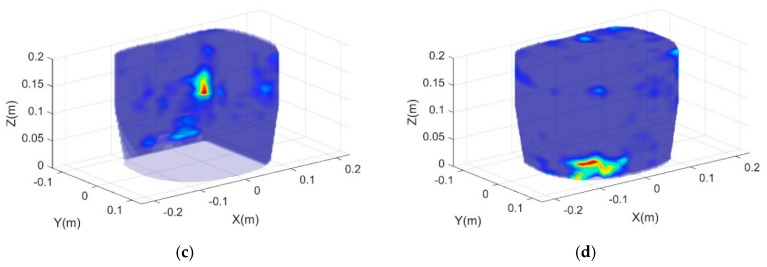
(**a**) Water located inside a plastic bag and its size is compared to the phantom lungs. (**b**) Two different positioning that water was located inside the lungs. (**c**) Constructed image for case 1, where water is located at rear side of the left lung, and (**d**) case 2, where water is located at the lower side of the right lung.

An “unhealthy” case is created by physically positioning 30 mL of water inside one of the lung phantoms. Water, which has a measured permittivity in the range 84.4–83.8 F/m and conductivity of 0.2–0.9 S/m across the band 0.9 GHz–2 GHz, was used as target in the measurements. The volume of fluid used in these studies does not represent any special category of the disease. It was merely selected to determine the accuracy of the technique. The obtained raw signals in similar arbitrary positions are depicted in Figure 7c for the simulated diseased scenario. As realized, there is a difference between the level of obtained signals for each position between the empty domain and the diseased phantom. This difference is used to define any existing abnormality. The target, water, is located inside a plastic bag and is shown in Figure 8a. It is then positioned in two random places; (1) at the rear side of the left lung (Figure 8b) in a vertical alignment and (2) beneath the right-side lung (Figure 8b) in a horizontal alignment. These two positions are selected to examine the shape prediction capability of the system. It should be noted that the lungs and abdomen block are located inside the torso during all measurements. As shown in Figure 8c,d, by processing the scattered signals within the frequency range of 0.9–2 GHz using the imaging algorithm, the located target is detected. It can be seen that due to three-dimensional representation of the target, its spatial location is distinguished between these two different cases. To create the three-dimensional images, the two-dimensional images of the 23 vertical positions are interpolated using Equation (4) to enhance the three-dimensional image resolution to 2 mm by creating 115 slices. In a clinical scenario, this spatial location is necessary to accurately insert needles into the area of pathology for diagnostic aspiration or biopsy, or for therapeutic interventions. Currently, US and CT, and MRI to a lesser extent are used for this purpose. In addition, the imaging algorithm allows determination of the size and shape of the embedded target. The constructed image for case 1, Figure 8c, shows a target has a thickness across z-axis, whereas the image for case 2, Figure 8d, shows a distribution of fluid across *x*- and *y*-axis. This is valuable in a clinical situation to monitor for changes in fluid collection to monitor potentially deteriorating situations (such as bleeding into a lung) and gauge response to therapeutic interventions (such as shrinking of an abscess in response to antibiotics and/or drainage procedures). It is noted that CT-scan and US are frequently used for this purpose, but not in real time, which is the advantage of the proposed system.

## 5. Conclusions

An electromagnetic torso scanner that is capable of generating 3D images is designed, fabricated and verified. The system was successfully tested to identify a chest-related disease, e.g., pulmonary abscess. The system is capable of showing the location and volume of the target in an imaged area, such as the chest. Considering the low cost of fabrication, the portability of the system, and its simple, safe and non-ionizing mechanism, this device has the potential to be utilized as a diagnostic tool to perform initial screening on patients in medical clinics or in mobile units such as ambulances. Due to its volumetric representation of the target, it can be used as a tool to monitor patient progress.
